# Outcomes Between Stemmed and Stemless Total Shoulder Arthroplasty: A Systematic Review and Meta-analysis of Randomized Controlled Trials

**DOI:** 10.5435/JAAOSGlobal-D-22-00077

**Published:** 2022-11-02

**Authors:** Austin M. Looney, Jonathan Day, John L. Johnson, Peter S. Johnston

**Affiliations:** From the Department of Orthopaedic Surgery, Georgetown University Hospital, Washington, DC (Dr. Looney, Dr. Day, and Dr. Johnson), and the Southern Maryland Orthopaedic & Sports Medicine Center, Centers for Advanced Orthopaedics, Leonardtown, MD (Dr. Johnston).

## Abstract

Both stemmed and stemless designs for total shoulder arthroplasty (TSA) have demonstrated efficacious outcomes for the surgical treatment of primary glenohumeral joint osteoarthritis. The purpose of this systematic review and meta-analysis was to compare the clinical outcomes of stemmed versus stemless TSA in randomized controlled trials. We hypothesized that there would be no differences in Constant Score (CS), range of motion, or adverse events, such as periprosthetic fracture and/or revision surgery.

**Methods:**

Using the Preferred Reporting Items for Systematic Reviews and Meta-analyses guidelines, a systematic review of the literature was done using MEDLINE, SPORTDiscus, Cumulative Index to Nursing and Allied Health Literature, Cochrane Central Registry of Controlled Trials, Embase, and Web of Science databases. Outcomes of interest included CS, range of motion, and adverse events (periprosthetic fracture and revision). Summary effect estimates of the mean difference between stemmed and stemless TSA for each outcome were estimated in random effects models.

**Results:**

The search yielded 301 articles with 4 appropriate for qualitative analysis, including the results of 229 stemmed and 358 stemless TSAs. No significant difference was observed in postoperative CS (*P* = 0.36), forward flexion (*P* = 0.93), abduction (*P* = 0.30), or external rotation (*P* = 0.34) between stemmed and stemless TSA. No significant difference was observed in change in CS (*P* = 0.27), forward flexion (*P* = 0.25), or external rotation (*P* = 0.74). A change in abduction was significantly different between stemmed and stemless TSA (standardized mean difference = −0.64; 95% confidence interval, −1.20 to −0.08) in favor of stemmed TSA (*P* = 0.02), attributed to preoperative differences. No significant difference was observed in periprosthetic fractures (*P* = 0.07) or revision (*P* = 0.90).

**Conclusion:**

TSA with stemless versus stemmed humeral components was not associated with notable differences in functional and clinical outcomes. No difference was observed between stemmed and stemless designs in postoperative forward flexion, abduction, or external rotation. Similarly, there was no difference in change in forward flexion or external rotation. A markedly greater improvement in abduction was observed with stemmed TSA, likely due to the lower preoperative motion in the stemmed cohort in one of the studies. No differences were observed between stemmed and stemless designs in the rate of humeral fracture or risk of revision.

**Level of Evidence:**

Level II; systematic review and meta-analysis of prospective randomized controlled trials.

Stemmed total shoulder arthroplasty (TSA) has been considered the benchmark surgical treatment for primary glenohumeral osteoarthritis (PGHOA) with intact, functional rotator cuff.^1-3^ Although stemmed TSA yields favorable clinical and radiographic outcomes, there are several potential complications associated with this implant design.^4,5^ Specifically, complications attributed to the humeral stem implant, such as periprosthetic fractures, loss of bone stock and osteolysis, malalignment, and stress-shielding, have been reported in the literature.^6,7,8,9,10,11^

This concern has led to the advent of newer implant designs featuring a stemless humeral implant to minimize the potential risks. Subsequent short-term to midterm follow-up studies of these stemless TSA designs have reported promising clinical and radiographic outcomes, with several newer head-to-head comparisons reporting outcomes and complications in stemmed and stemless designs.^12,13,14,15^ However, the levels of evidence (LoE) in these comparative studies are limited because of small sample sizes and study designs. In our experience, the short-term outcomes of both implant designs are comparable. Several prospective randomized trials have emerged in the literature,^16,17,18,19^ and although these studies provide stronger evidence, an in-depth analysis aggregating these results is needed.

Therefore, the purpose of this systematic review and meta-analysis was to compare the short-term clinical and radiographic outcomes in level I and II prospective randomized trials in which patients were randomized to stemmed or stemless TSA for the treatment of PGHOA. We hypothesized that there would be no differences observed in functional range of motion (ROM), age-related and gender-related Constant Score (CS), or adverse events such as periprosthetic fractures and/or revision surgery between stemmed and stemless designs.

## Methods

### Protocol and Registration

This systematic review and meta-analysis was conducted according to the Preferred Reporting Items for Systematic Reviews and Meta-analyses guidelines and checklist.^20^ A protocol was registered with PROSPERO (National Institute for Health Research) published on January 18, 2021 (ID: CRD42021227136).

### Search Strategy

A systematic review of the literature was done using MEDLINE, SPORTDiscus, Cumulative Index to Nursing and Allied Health Literature, Cochrane Central Registry of Controlled Trials, Embase, and Web of Science databases. This search included all available literature up to January 2021. The following terms were used: “total shoulder arthroplasty,” “stemmed,” “stemless,” “shoulder replacement,” and “shoulder prosthesis” combined with the Boolean operators AND/OR.

### Study Selection

English-language prospective randomized trials reporting at least one of the outcomes of interest (CS, ROM, and/or revisions) for the treatment of PGHOA with stemmed and stemless TSA were screened as potentially eligible, and nonduplicate articles were evaluated independently by two reviewers (A.M.L. and J.D.) for inclusion. To be included in the analysis, the following additional criteria had to be met: (1) stated implant design (i.e., stemmed versus stemless TSA) and (2) outcomes stratified by implant design. Grounds for exclusion included the following: (1) minimum follow-up 24 months, (2) LoE other than I or II (i.e., not a prospective randomized trial), (3) no outcomes of interest reported in a way that would allow comparison through quantitative methods, and (4) incomplete/ongoing trials. Two authors (A.M.L. and J.D.) assessed all full-text articles to verify appropriateness for inclusion/exclusion. Disagreements were resolved to consensus through discussion.

### Data Extraction

Two authors (A.M.L. and J.D.) independently reviewed each study and collected the following: year of publication, LoE in accordance with the American Academy of Orthopaedic Surgeons guidelines,^21^ number of cases and patients, method of treatment (stemmed/stemless TSA), and follow-up period. Demographic information such as mean age and sex distribution was recorded. The following functional and clinical outcomes were extracted: CS, shoulder ROM (forward flexion, abduction, and external rotation), and postoperative complications (incidence of periprosthetic fracture and revision surgery). CSs were reported using the same methodology developed by Constant and Murley.^22^ Radiographic outcomes were collected and reported descriptively because of the inconsistent nature of reporting across the studies and therefore was not appropriate for inclusion for meta-analysis.

### Risk of Bias in Individual Studies

The risk of bias in individual studies was assessed using a validated Cochrane risk assessment tool specifically designed for randomized controlled trials (Cochrane RoB2 tool).^23^ The tool uses a series of questions to screen for features relevant to risk of bias. Studies were evaluated by two of the authors (A.M.L. and J.D.), and each domain was scored as low risk of bias, high risk of bias, or unclear risk of bias. Any disagreements were resolved through discussion to consensus.

### Risk of Bias Across Studies

Funnel plot analysis was done to assess for risk of publication bias. The plot was inspected visually for symmetry and analyzed with the trim-and-fill method.^24,25^ The Begg and Egger tests were also conducted.^26,27^

### Synthesis of Results

Study-level effect sizes consisted of means and SDs for the outcomes of interest. Due to the variety of implant manufacturers across the included studies and additional surgical and technical variations that are possible between surgeons and institutions but difficult to account for, we obtained estimates of summary effect using random effects models with the restricted maximum likelihood method. Summary effects were estimated as the standardized mean difference in change (i.e., from preoperative to postoperative assessments) and the mean difference in postoperative score between stemmed and stemless TSA. Separate models were evaluated for CS, forward flexion, abduction, external rotation, and revision surgery incidence. Heterogeneity was evaluated with Cochrane's *Q*-test, *τ*^2^, and *I*^2^. *I*^2^ values of 0% to 35%, 35% to 65%, 65% to 85%, and 85% to 100% were considered low, moderate, substantial, and considerable levels of heterogeneity, respectively.^28^ All analysis was conducted in R (v3.6.3, R Foundation for Statistical Computing) by an author with experience in previous orthopaedic meta-analyses (A.M.L.), with the “meta” and “metafor” packages.^29,30^ The criterion for statistical significance was *P* < 0.05.

### Additional Analysis

Since implant manufacturers differed between the included studies, we assessed the relationship between manufacturer and CS outcomes in mixed effects models with a fixed-effects moderator. In mixed-effects models with moderators, *Q*_*E*_ represents a test for residual heterogeneity (variability in the observed effect sizes not accounted for by the moderators), and *Q*_*M*_ represents an omnibus test for observed effect size variability accounted for by all model coefficients.^30^

## Results

### Study Selection

**Figure 1:**
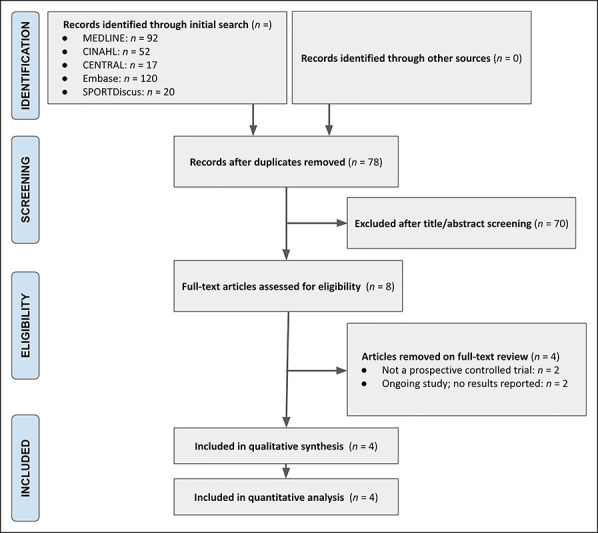
Flowchart showing the PRISMA article selection diagram. PRISMA = preferred reporting of information for meta-analysis

The initial search yielded 301 articles. On the full-length review, four articles were appropriate for inclusion in the qualitative and quantitative analysis.^16,17,18,19^ The Preferred Reporting Items for Systematic Reviews and Meta-analyses selection process is illustrated in Figure 1.

### Study Characteristics

The four included articles consisted of two level I studies^17,18^ and two level II studies.^16,19^ The surgical indication for TSA was similar for all patients in all four studies, namely PGHOA with intact rotator cuff function. Randomization in the studies was achieved using calendar date of operation,^17^ computer-automated randomization,^19^ and site-by-site block randomization.^18^ The randomization method was not reported in one study.^16^

Stemmed implant designs included the Univers II (Arthrex),^16,19^ Aequalis,^17^ and Comprehensive Mini (Zimmer Biomet).^18^ Stemless designs included the Eclipse (Arthrex),^16,19^ Aequalis,^17^ and Comprehensive Nano (Zimmer Biomet).^18^ In all four studies, surgical exposure was achieved through a deltopectoral approach.^16,17,18,19^ Subscapularis tenotomy with tendon-to-tendon reattachment was used exclusively in one study,^16^ while a combination of subscapularis tenotomy and lesser tuberosity osteotomy was done based on surgeon preference in the remaining three studies.^17-19^

The use of cementing and fixation was done according to manufacturer's specifications.^17-19^ The glenoid implant was usually cemented,^16-18^ while the humeral implant was noncemented in both stemmed and stemless designs.^16-18^ Postoperative rehabilitation in all studies consisted of immobilization in 10° to 30°^16,17^ of abduction in a shoulder orthosis for the first 2 to 4 weeks,^16,17,19^ with passive, limited ROM, progressing to active and passive ROM without restrictions after 6 weeks.

Additional characteristics are summarized in Table 1. The specific postoperative protocols as provided by the included studies are detailed in Table 2.

**Table 1:** Characteristics of the Included Studies

Study						Stemmed TSA				Stemless TSA			
Author	Journal	Year	LoE	Implant manufacturer	Follow-up (mo)	*n*	Age^a^	M:F	Implant^b^	*n*	Age	M:F	Implant^b^
Mariotti	*Musculoskelet Surg*	2014	I	Tornier	24	10	NR	NR	Aequalis stemmed	9	NR	NR	Aequalis stemless
Romeo	*JSES*	2020	II	Arthrex	24	78	66^c^	57:21	Univers II	218	66^a^	151:67	Eclipse
Uschok	*JSES*	2017	II	Arthrex	24	18	69^d^	7:13	Univers II	15	65	10:10	Eclipse
Wiater	*JBJS*	2020	I	Zimmer	24	123	62.1 ± 9.6^e^	87:46	Comprehensive mini	116	63.1 ± 9	89:43	Comprehensive nano

LoE = level of evidence, M:F = male-to-female ratio, NR = not reported, TSA = total shoulder arthroplasty

a Reported in years.

b Humeral implant.

c Median.

d Average.

e Mean ± SD.

**Table 2:** Postoperative Protocols for TSA

Study^a^	Immobilization		Therapy/rehabilitation	
	Device	Duration	Protocol	Time initiated
Mariotti (2014)	10° abduction orthosis	1 month	PROM only Pendulums starting week 3 AROM: ● Forward flexion to 90° ● Abduction to 90° ● External rotation to neutral No additional restrictions	Immediate Week 3 Week 5 Week 6
Romeo (2020)	Sling	NR	PROM only “Progressed to active-assisted and then active motion” Strengthening No resisted internal rotation	Immediate NR Full ROM achieved 12 weeks
Uschok (2017)	30° abduction brace	3 weeks	Limited PROM Unrestricted AROM/PROM + strengthening	Immediate Week 7
Wiater (2020)	Sling	3–4 weeks	Phase 1: PROM ● Supine forward flexion to tolerance ● External rotation in scapular plane to 30° ● Internal rotation to chest Phase 2: Early strengthening ● Begin AROM (forward flexion, abduction, external/internal rotation in the scapular plane) ● AAROM pulleys (forward flexion, abduction) ● Scapular strengthening Phase 3: Moderate strengthening ● Progress AROM as appropriate ● Advance PROM to stretching as appropriate ● Assisted internal rotation behind back stretch ● Resisted external/internal rotation Return to recreational sports	Immediate Weeks 4 to 6 Week 6 4-6 months postop

AAROM = assisted active range of motion, AROM = active range of motion, NR = not reported, PROM = passive range of motion, ROM = range of motion

a Lead author last name (year of publication).

### Risk of Bias Within Studies

**Figure 2:**
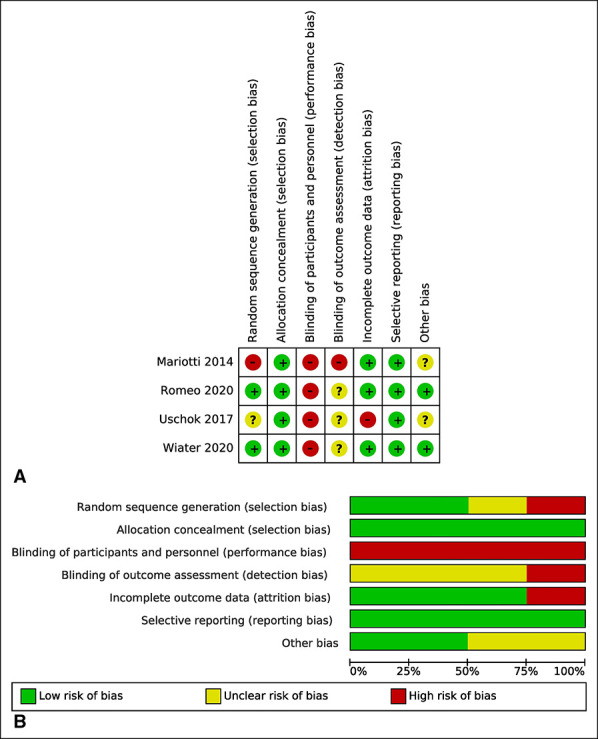
Diagram showing the Cochrane risk of bias assessment.

All four studies were rated as being high risk for performance bias, given that in all cases, the surgeon performing the operations knew (at the time of the operation) which implant he was using for each patient, although this is probably unavoidable. The studies were also not clear regarding the degree to which outcomes assessors were blinded to the choice of implant (detection bias). Otherwise, the studies were deemed to be reasonably low-risk for bias in the remaining domains (Figure 2).

### Risk of Bias Across Studies

**Figure 3:**
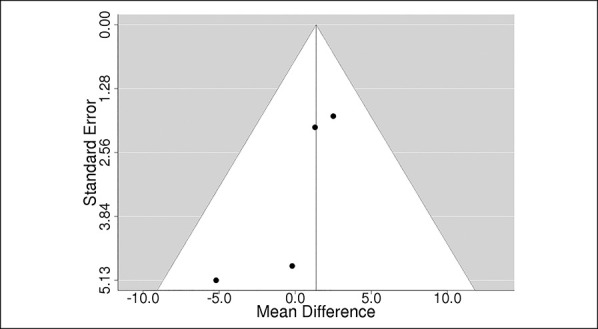
Diagram showing the funnel plot analysis for publication bias.

A funnel plot was constructed to assess for publication bias (Figure 3). The Begg and Egger tests were insignificant (*P* = 0.083 and *P* = 0.204, respectively).

### Synthesis of Results

The four studies randomized a total of 634 shoulders in 620 patients (403 [65%] men, 217 [35%] women) and included the 2-year results for a total of 358 stemless humeral implants and 229 stemmed humeral implants. Summary statistics for average age across studies could not be determined because one of the studies did not provide this information.^17^

### Constant Score

**Figure 4:**
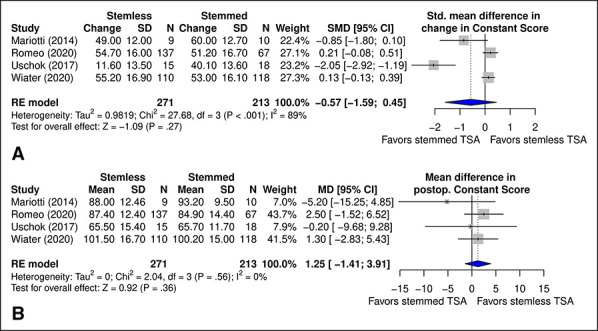
Diagram showing the comparison of (**A**) SMD in change in CS and (**B**) MD in postoperative CS between total shoulder arthroplasty with stemmed and stemless humeral implants. CI = confidence interval, CS = constant source, MD = mean difference, SMD = standard mean difference, TSA = total shoulder arthroplasty

No significant difference was observed in change in CS (*P* = 0.27) (Figure 4, A) or postoperative CS (*P* = 0.36) (Figure 4, B) between TSA with stemmed and stemless humeral implants.

CS (reported by all included studies) ranged from 25.6 ± 15.2 to 47.2 ± 17.1^18^ preoperatively and 65.7 ± 11.7^16^ to 100.2 ± 15.0^18^ postoperatively in stemmed TSA. In stemless TSA, CS ranged from 32.4 ± 11.1^19^ to 53.9 ± 11.3^16^ preoperatively and 65.5 ± 15.4^16^ to 101.5 ± 16.7^18^ postoperatively.

### Range of Motion

#### Forward Flexion

**Figure 5:**
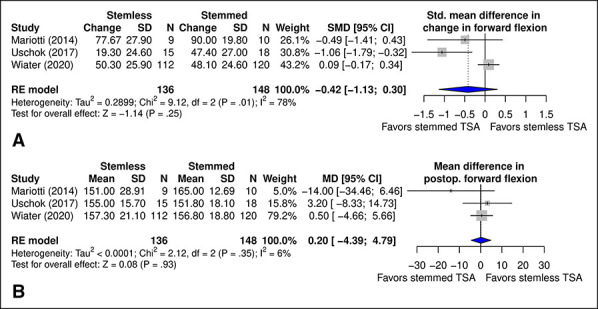
Diagram showing the comparison of (**A**) SMD in change in forward flexion and (**B**) MD in postoperative forward flexion between TSA with stemmed and stemless humeral implants. CI = confidence interval, MD = mean difference, SMD = standard mean difference, TSA = total shoulder arthroplasty

No significant difference was observed in change in forward flexion between stemmed and stemless TSA (*P* = 0.25) (Figure 5. A). Similarly, there was no significant difference in postoperative forward flexion (*P* = 0.93) (Figure 5, B).

Forward flexion was reported in 3^16-18^ studies, ranging from 75° ± 25°^17^ to 108.7° ± 29.3°^18^ preoperatively and 151.8° ± 18.1°^16^ to 165° ± 12.7°^17^ postoperatively in stemmed TSA. In stemless TSA, forward flexion ranged from 73.3° ± 26.9°^17^ to 135.7° ± 31.1°^16^ preoperatively and 151° ± 28.9°^17^ to 157.3° ± 21.1°^18^ postoperatively.

#### Abduction

**Figure 6:**
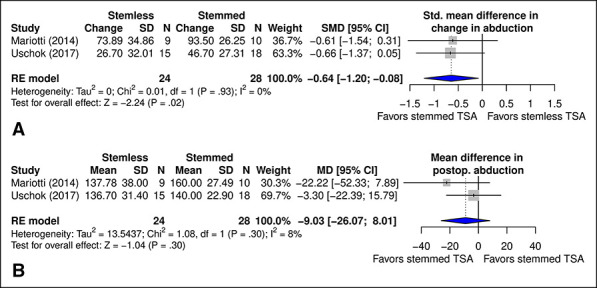
Diagram showing the comparison of (**A**) SMD in change in abduction and (**B**) MD in postoperative abduction between TSA with stemmed and stemless humeral implants. CI = confidence interval, MD = mean difference, SMD = standard mean difference, TSA = total shoulder arthroplasty

Change in abduction was significantly different between stemmed and stemless TSA (standardized mean difference = −0.64; 95% confidence interval [CI], −1.20 to −0.08) (Figure 6, A) in favor of stemmed TSA (*P* = 0.02); however, there was no significant difference in postoperative abduction (*P* = 0.30) (Figure 6, B).

Abduction was reported in 2^16,17^ studies, ranging from 66.5° ± 24.9°^17^ to 93.3° ± 31.1°^16^ preoperatively and 140° ± 22.9°^16^ to 160° ± 27.5°^17^ postoperatively in stemmed TSA. In stemless TSA, abduction ranged from 63.9° ± 31.4°^17^ to 110° ± 32.6°^16^ preoperatively and 136.7° ± 31.4°^16^ to 137.8 ± 38.0°^17^ postoperatively.

#### External Rotation

**Figure 7:**
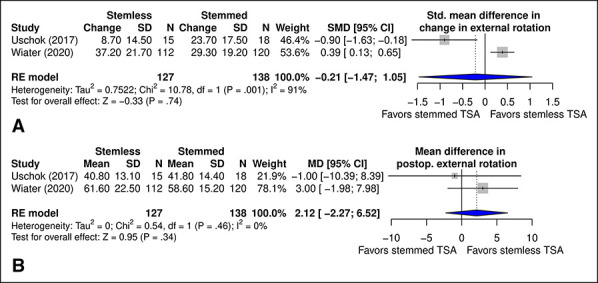
Diagram showing the comparison of (**A**) SMD in change in external rotation and (**B**) MD in postoperative external rotation between TSA with stemmed and stemless humeral implants. CI = confidence interval, MD = mean difference, SMD = standard mean difference, TSA = total shoulder arthroplasty

No significant difference was observed between stemmed and stemless designs in change in external rotation (*P* = 0.74) (Figure 7, A) or postoperative external rotation (*P* = 0.34) (Figure 7, B).

External rotation was reported in 2^16,18^ studies, ranging from 18.1° ± 20.1°^16^ to 29.3° ± 22.5°^18^ preoperatively and 41.8° ± 14.4°^16^ to 58.6° ± 15.2°^18^ postoperatively in stemmed TSA. In stemless TSA, external rotation ranged from 24.4° ± 20.8°^18^ to 32.1° ± 15.8°^16^ preoperatively and 40.8° ± 13.1°^16^ to 61.6° ± 22.5°^18^ postoperatively.

### Adverse Events

#### Humerus Fractures

**Figure 8:**
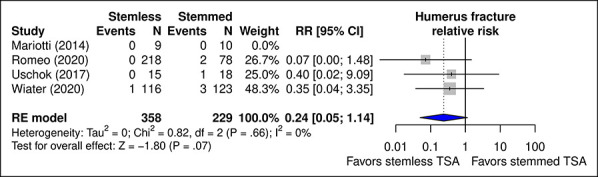
Diagram showing the RR of humerus fracture with stemmed versus stemless implants for TSA. CI = confidence interval, RR = relative risk, TSA = total shoulder arthroplasty

Fewer fractures were reported for TSA with stemless implants, but the difference was not significant (*P* = 0.07) (Figure 8). Only one fracture was reported postoperatively;^16^ the remainder occurred and were managed intraoperatively. The proportion of cases complicated by periprosthetic fractures ranged from 0%^17^ to 5.6%^16^ for stemmed TSA and from 0%^16,17,19^ to 0.9%^18^ for stemless TSA.

#### Revision

**Figure 9:**
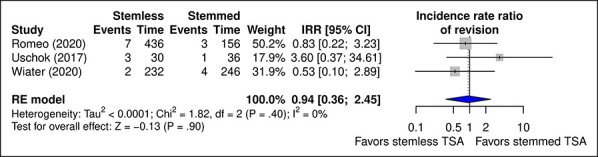
Diagram showing the IRR of revision surgery with stemmed versus stemless TSA. Exposure represents person-years of follow-up. CI = confidence interval, IRR = incidence rate ratio, TSA = total shoulder arthroplasty

No significant difference was observed between stemmed and stemless TSA in the relative risk of revision (*P* = 0.90) (Figure 9). The incidence of revised cases with stemmed TSA ranged from 3.3%^18^ to 5.6%^16^ and from 1.7%^18^ to 3.2%^19^ with stemless TSA.

### Radiographic Findings

Regarding the radiographic results, one study reported lower bone density surrounding the humeral head in 66.7% of stemmed TSA compared with 28.6% of stemless TSA.^16^ No radiolucent lines were observed in the remaining studies in both stemmed and stemless cohorts. Additional study findings and outcomes are summarized in Tables 3 and 4.

**Table 3:** Outcomes and Clinical Findings

Study^a^	Patient-reported outcomes^b^	Motion^b,c^	Radiographic	Conclusions
Mariotti (2014)	CS ● Stemmed: 33.2 ± 15.32 → 93.2 ± 9.50 ● Stemless: 39.0 ± 11.61 → 88.0 ± 12.46 ● Stemmed versus stemless: No significant difference in preop (*P* = 0.278) or postop (*P* = 0.170) scores SST ● Stemmed: 1.1 ± 1.37 → 10.5 ± 2.27 ● Stemless: 1.67 ± 1.58 → 9.67 ± 2.45 ● Stemmed versus stemless: No significant difference in preop (*P* = 0.373) or postop (*P* = 0.307) scores	Forward flexion ● Stemmed: 75.0° ± 25.0° → 165° ± 12.69° ● Stemless: 73.33° ± 26.92° → 165° ± 12.69° ● Stemmed versus stemless: No significant difference in preop (*P* = 0.833) or postop (*P* = 0.319) motion Abduction ● Stemmed: 66.5° ± 24.94° → 160° ± 27.49° ● Stemless: 63.89° ± 31.40° → 137.78° ± 38° ● Stemmed versus stemless: No significant difference in preop (*P* = 0.905) or postop (*P* = 0.152) motion Internal rotation (score**)** ● Stemmed: 2.0° ± 2.54° → 5.4° ± 1.35° ● Stemless: 2.25° ± 1.82° → 5.55° ± 1.67° ● Stemmed versus stemless: No significant difference in preop (*P* = 0.675) or postop (*P* = 0.613) scores External rotation (score**)** ● Stemmed: 6.0° ± 8.09° → 51.5° ± 16.17° ● Stemless: 10.00° ± 9.01° → 45.55° ± 18.1° ● Stemmed versus stemless: No significant difference in preop (*P* = 0.345) or postop (*P* = 0.335) scores	No evidence of loosening detected for either implant by the 2-year follow-up.	TSA with stemmed and stemless humeral components resulted in comparable ROM and similar functional outcomes at 2 years.
Romeo (2020)	CS ● Stemmed: 33.4 ± 11.5 → 84.9 ± 14.43 ● Stemless: 2.4 ± 11.1 → 87.4 ± 12.4 ● Stemmed versus stemless: No significant difference in preop (*P* = 0.501) or postop (*P* = 0.203) scores CCS score ● Stemmed: 89.7% success ● Stemless: 92.3% success ● *H*_*0*_ of stemless inferiority rejected^d^ Visual analog scale (pain) ● *“No significant differences in overall VAS pain score or degree of change in VAS pain score from baseline”* ● Actual data NR 36-Item short-form survey ● *“No significant differences existed between the groups regarding overall SF-36 mental health implant or physical function implant scores or change in SF-36 mental health implant or physical function implant scores from baseline”* ● Actual data NR	Forward flexion^e^ ● Stemmed: NR ● Stemless: NR ● Stemmed versus stemless: NR Abduction^e^ ● Stemmed: NR ● Stemless: NR ● Stemmed versus stemless: NR Internal rotation^e^ ● Stemmed: NR ● Stemless: NR ● Stemmed versus stemless: NR External rotation^e^ ● Stemmed: NR ● Stemless: NR ● Stemmed versus stemless: NR	Humeral implants ● Radiolucency: None ● Subsidence: None ● Migration: None Glenoid implants ● Radiolucency: None > grade 3 ● Subsidence: None ● Migration: None	Stemless TSA is safe and effective for patients with PGHOA at the 2-year follow-up, with no differences in outcomes compared with stemmed TSA by the same manufacturer.
Uschok (2017)	CS ● Stemmed: 25.6 ± 15.2 → 65.7 ± 11.7 ● Stemless: 53.9 ± 11.3 → 65.5 ± 15.4 ● Stemmed versus stemless: No significant difference in postop scores (*P* = 0.642) CS, pain subcategory ● Stemmed: 3.6 ± 2.3 → 13.6 ± 2.9 ● Stemless: 8.2 ± 3.5 → 10.9 ± 4.4 ● Stemmed versus stemless: No significant difference in postop score (*P* = 0.136) CS, ADL subcategory ● Stemmed: 4.6 ± 3.3 → 15.8 ± 4.2 ● Stemless: 11.3 ± 4.7 → 15.2 ± 4.6 ● Stemmed versus stemless: No significant difference in postop score (*P* = 0.689) CS, ROM subcategory ● Stemmed: 14.3 ± 8.7 → 28.6 ± 6.3 ● Stemless: 25.5 ± 4.7 → 32.7 ± 6.2 ● Stemmed versus stemless: No significant difference in postop score (*P* = 0.053) CS, strength subcategory ● Stemmed: 3.1 ± 3.0 → 5.9 ± 4.5 ● Stemless: 4.7 ± 3.1 → 5.9 ± 3.9 ● Stemmed versus stemless: No significant difference in postop score (*P* = 0.912)	Forward flexion ● Stemmed: 104.4° ± 33.7° → 151.8° ± 18.1° ● Stemless: 135.7° ± 31.1° → 155.0° ± 15.7° ● Stemmed versus stemless: No significant difference in postop motion (*P* = 0.615) Abduction ● Stemmed: 93.3° ± 31.1° → 140° ± 22.9° ● Stemless: 110.0° ± 32.6° →136.7° ± 31.4° ● Stemmed versus stemless: No significant difference in postop motion (*P* = 0.982) Internal rotation ● Stemmed: NR ● Stemless: NR ● Stemmed versus stemless: NR External rotation ● Stemmed: 18.1° ± 20.1° → 41.8° ± 14.4° ● Stemless: 32.1° ± 15.8° → 40.8° ± 13.1° ● Stemmed versus stemless: No significant difference in postop motion (*P* = 0.845)	Humeral implants ● Radiolucency: Significantly greater in 1 of 3 zones (below caudal trunnion) with stemmed implants (*P* = 0.047), without influence on functional outcome ● Migration: Cranial migration of humeral head in 2 (14.3%) stemless TSAs versus 4 (23.5%) stemmed TSAs (*P* = 0.619); all clinically irrelevant Glenoid implants ● Radiolucency: No significant difference between stemmed versus stemless (*P* = 0.619)	Consistent functional outcomes at the 5-year follow-up with stemmed and stemless TSA, though without significant improvement from 2 to 5 years. No evidence of increased loosening with stemless designs. Increased radiolucency occurred around the caudal trunnion with the stemmed implant, probably due to stress shielding, but did not seem to affect functional outcomes.
Wiater (2020)	CS^f^ ● Stemmed: 45.0 (34.0-60.0) → 102.0 (92.0-111.0) ● Stemless: 45.0 (34.0-57.0) → 104.5 (94.0-111.0) ● Stemmed versus stemless: No significant difference in preop (*P* = 0.78) or postop (*P* = 0.31) scores ASES score^f^ ● Stemmed: 28.3 (18.3-33.3) → 98.3 (91.7-100.0) ● Stemless: 28.3 (16.7-34.2) → 98.3 (92.5-100.0) ● Stemmed versus stemless: No significant difference in preop (*P* = 0.76) or postop (*P* = 0.45) scores SANE score^f^ ● Stemmed: 51.0 (40.0-75.0) → 95.0 (90.0-100.0) ● Stemless: 50.0 (40.0-75.0) → 98.0 (90.0-100.0) ● Stemmed versus stemless: No significant difference in preop (*P* = 0.46) or postop (*P* = 0.65) scores	Forward flexion ● Stemmed: 108.7 ± 29.3 → 156.8 ± 18.8 ● Stemless: 107.0 ± 30.0 → 157.3 ± 21.1 ● Stemmed versus stemless: No significant difference in preop (*P* = 0.64) or postop (*P* = 0.85) motion Abduction ● Stemmed: NR ● Stemless: NR ● Stemmed versus stemless: NR Internal rotation ● Stemmed: NR ● Stemless: NR ● Stemmed versus stemless: NR External rotation ● Stemmed: 29.3 ± 22.5 → 58.6 ± 15.2 ● Stemless: 24.4 ± 20.8 → 61.6 ± 22.5 ● Stemmed versus stemless: Stemless significantly lower preop (*P* = 0.06); no significant difference postop (*P* = 0.25)	Humeral implants ● Radiolucency: > 2 mm confined to 1 zone in 2 stemmed implants; none in stemless implants ● Subsidence: None ● Migration: None Glenoid implants ● Radiolucency: None ● Subsidence: None ● Migration: None	Stemless humeral TSA implant demonstrated noninferior safety and effectiveness compared with stemmed implant. Early results suggest potential benefits of stemless design.

ADL = activities of daily living, ASES = American Shoulder and Elbow Surgeons, CCS = composite clinical success, CS = constant source, NR = not reported, PGHOA = primary glenohumeral osteoarthritis, ROM = range of motion, SANE = single assessment numeric evaluation, SF-36 = short form 36, SST = simple shoulder test, TSA = total shoulder arthroplasty, VAS = visual analog scale

a Lead author last name (year of publication).

b Preoperative → postoperative, mean ± SD unless otherwise noted.

c Active.

d Corrected one-sided 98.131% CI lower bound = −6.4%; inferiority criterion ≤−10%.

e ^“^ROM was not consistently assessed…”.

f Median (interquartile range).

**Table 4:** Adverse Events

Study^a^	Humerus fracture					
	Intraoperative		Postoperative		Subsequent surgeries	
	Stemmed	Stemless	Stemmed	Stemless	Stemmed	Stemless
Mariotti (2014)	*n* = 0	*n* = 0	*n* = 0	*n* = 0	NR	NR
Romeo (2020)	*n* = 2	*n* = 0	*n* = 0	*n* = 0	*n* = 3 (3.8%) Infection Distal clavicle excision (*n* = 1) Conversion to RTSA (*n* = 2)	*n* = 7 (3.2%) Infection (*n* = 3) Conversion to RTSA (*n* = 3) Subscapularis repair (*n* = 1)
Uschok (2017)	*n* = 0	*n* = 0	*n* = 1	*n* = 0	*n* = 1 (6.7%) Loosening from greater tuberosity fracture	*n* = 3 (7.1%) Atraumatic glenoid loosening (n = 2) Conversion to RTSA (*n* = 1)
Wiater (2020)	*n* = 1	*n* = 0	*n* = 2	*n* = 1	*n* = 4 (3.3%) Infection (*n* = 2) Conversion to RTSA (*n* = 2)	*n* = 2 (1.7%) Infection (*n* = 2)

NR = not reported, RTSA = reverse total shoulder arthroplasty

a Lead author last name (year of publication).

### Additional Analysis: Influence of Implant Manufacturer

**Figure 10:**
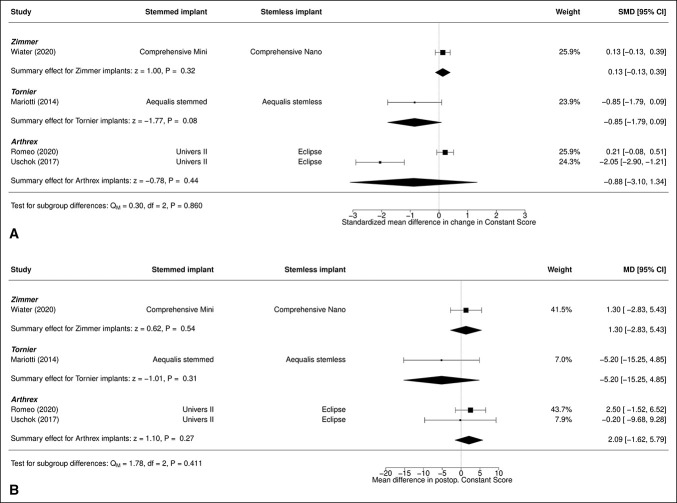
Mixed effects model analysis for the effect of implant manufacturer on (**A**) SMD in change in CS or (**B**) MD in postoperative CS. CI = confidence interval, CS = constant source, MD = mean difference, SMD = standard mean difference

Among the analyzed studies, two used Arthrex implants,^16,19^ one used Tornier implants,^17^ and one used Zimmer Biomet implants.^18^ Implant manufacturer was not significantly associated with any difference in change in CS (*P* = 0.860) or postoperative score (*P* = 0.411) (Figure 10). Linear combinations of the various manufactures did not reveal any significant differences between any two brands (Table 5).

**Table 5:** Effect of TSA Implant Manufacturer on CS Outcomes

Contrast^a^	SMD, Change in CS					MD, Postoperative CS				
	Est.^b^	SE	95% CI	*Q*_*M*_	*P* Value	Est.^b^	SE	95% CI	*Q*_*M*_	*P* Value
Arthrex versus Tornier	−0.03	1.99	−3.94 to 3.87	0.00	0.987	7.29	5.46	−3.42 to 18.00	1.78	0.182
Arthrex versus Zimmer	−1.02	1.94	−4.82 to 2.79	0.27	0.601	0.79	2.83	−4.67 to 6.34	0.08	0.781
Tornier versus Zimmer	−0.98	2.27	−5.44 to 3.48	0.19	0.666	−6.50	5.54	−17.36 to 4.36	1.38	0.241

CI = confidence interval, CS = constant source, Est. = estimate, MD = mean difference, SE = standard error, SMD = standardized MD, TSA = total shoulder arthroplasty

a Linear combinations of categorical coefficients. Each contrast represents a hypothesis test of the form *β*_1_ − *β*_2_ = 0.

b Result of the linear combination.

## Discussion

The main finding of this analysis was that there were no notable differences in change in CS or postoperative CS between TSA with stemmed and stemless humeral implants. In addition, we observed no difference in postoperative ROM or incidence of periprosthetic fractures or revision surgery. Our meta-analysis included a total of 634 shoulders in 620 patients with 358 stemless implants and 229 stemmed implants and represents the largest such study to date comparing outcomes of TSA with stemmed and stemless humeral implants from high-quality (evidence levels I and II) prospective randomized studies. Our findings support the hypothesis that there is no difference in results of TSA with stemmed or stemless designs.

### Functional Outcomes

CS was the primary measure of functional outcome examined by the present analysis and was consistently reported by the four included studies. No notable difference was observed between stemmed and stemless TSA in change in CS or in postoperative scores. Additional functional outcome assessments reported by these studies included the American Shoulder and Elbow Surgeons score; Single Assessment Numeric Evaluation score; Composite Clinical Success score, Visual Analog Scale, and Short form 36; and Simple Shoulder Test score. Uschock et al^16^ reported only CS; Wiater et al^18^ reported CS, American Shoulder and Elbow Surgeons, and Single Assessment Numeric Evaluation; Mariatti et al^17^ reported CS and Simple Shoulder Test; and Romeo et al^19^ reported CS, Composite Clinical Success, Visual Analog Scale, and Short form 36. Although only the CS could be quantitatively compared, no differences were observed in other outcome instruments reported in the respective studies.

These findings are consistent with previous meta-analyses comparing CS between stemmed and stemless TSA and found no notable difference between designs.^31,32^ However, there are several methodological differences distinguishing this study. Peng et al^31^ used a fixed effects model, which does not statistically account for expected clinical heterogeneity (e.g., related to variations in surgical technique and experimental environment) like the random effects analyses in this study. In addition, our analysis represents a synthesis of the best available evidence, as we restricted inclusion to prospective randomized trials (i.e., evidence levels I and II), while Peng et al^31^ included results from retrospective studies. Liu et al^32^ used a random effects model in their CS analysis but included evidence level III and IV studies. Additional differences include our adverse events analyses examining specific event types consistently reported across our included studies (thus allowing for a more reliable quantitative summary estimate), consideration of manufacturer effects through metaregression, and the fact that all patients in the included studies had TSA for a primary diagnosis of PGHOA.

### Range of Motion

With the exception of abduction, there were no notable differences between stemmed and stemless TSA in change in motion or in postoperative motion. Greater improvement in abduction was observed with stemmed TSA, but no notable difference in postoperative abduction between stemmed and stemless designs. Possibly, this difference is related to the lower preoperative abduction among the stemmed group in the study by Uschok et al^16^ (93.3° ± 31.1° for stemmed, 110° ± 32.6° for stemless). Similarly, there was no notable difference between stemmed and stemless TSA in the risk of periprosthetic fracture or incidence of revision.

ROM improvement varied among the included studies, although previous summary estimates for change fall within these ranges.^32,33^ Liu et al^32^ systematically reviewed ROM after stemless anatomic TSA and reported weighted average improvements in forward flexion from 90° ± 16° to 142° ± 17°, abduction from 70° ± 16° to 130° ± 21°, and external rotation from 23° ± 10° to 47° ± 11°, which compared favorably with reviews of stemmed TSA outcomes. In a systematic review and meta-analysis of shoulder arthroplasties with stemless humeral components, Willems et al^33^ observed the mean postoperative ROM of 137° for forward flexion (mean gain of 46° [95% CI, 39° to 50°]), 122° for abduction (mean gain of 52° [95% CI, 42° to 62°]), and 44° for external rotation (mean gain of 22° [95% CI, 17° to 27°]); however, those summary estimates included the results of 382 hemiarthroplasties among the 1564 shoulders. The systematic review and meta-analysis of Peng et al^31^ found no notable differences in forward flexion, abduction, or external rotation between anatomic TSA with stemmed and stemless humeral components, consistent with our observations.

### Complications

We observed no differences in incidence of periprosthetic fractures or revision surgery between stemmed and stemless humeral implants. Regarding the overall complication rates, the individual included studies consistently reported similar results with stemmed and stemless designs. Mariotti et al^17^ observed no radiographic signs of loosening in any of their patients and reported no intraoperative or postoperative complications in either the stemmed or the stemless group. The overall complication rate in the study by Wiater et al^18^ was 8% for the stemless cohort and 7% for the stemmed cohort, with 18 total complications (9 in stemmed, 9 in stemless). Each of the complications was treated nonoperatively with satisfactory outcomes. Romeo et al^19^ reported a complication rate of 3.2% in the stemless cohort and 3.8% in the stemmed cohort. The overall complication rate in the study by Uschok et al^16^ was 13.8%, with a rate of 7.1% in the stemless group and 6.7% in the stemmed group. As observed by Liu et al,^32^ reporting of complications may vary between studies; therefore, we did not quantitatively compare the overall rates of complications.

Our findings parallel those of previous systematic reviews/meta-analyses and large database studies.^32-34^ A national database review by Rasmussen et al^34^ demonstrated a revision rates of 2.8% in 761 stemless arthroplasties and 2.6% in 4398 stemmed arthroplasties. Intraoperative fractures were not discussed, but periprosthetic humeral fracture was the indication for one revision in the stemmed group and none in the stemless group. Willems et al^33^ found an overall complication rate of 9.7% for stemless humeral implants in anatomic TSA and hemiarthroplasty combined, with 7 humerus fractures, 93 (6.0%) reoperations, and 79 (5.1% revisions). They determined that these rates were comparable with those reported in a comprehensive review of stemmed TSA.^9^ Similarly, the meta-analysis by Liu et al^32^ reported a complication rate of 8.3% in stemmed TSA most commonly due to rotator cuff failure (2.2%), infection (1%), glenoid loosening (0.06%), and glenoid perforation (0.08%). They observed no notable difference between overall complication rates with stemmed and stemless TSA from comparative studies but—as previously stated—noted inconsistency of reporting among studies as a limitation of this analysis. They determined an overall 5.6% rate of revision in stemless TSA—most commonly due to rotator cuff failure (1.8%), glenoid loosening (0.8%), and glenoid failure 0.8%).

### Radiographic Assessments

Meta-analysis of radiographic features (apart from periprosthetic fractures) after TSA was not undertaken in the present analysis because of the inconsistent nature of such data; however, various radiographic comparisons between stemmed and stemless implants were reported in the included studies. The study by Uschok et al^16^ examined the bone density of multiple radiographic points and humeral head migration to examine the effects of stress shielding and implant positioning, respectively. They noted partial lowering of the bone density surrounding the humeral head in 28.6% of the patients in the stemless group compared with 66.7% in the stemmed group, although this finding was not statistically significant.^16^ Regarding the glenoid implant, there was a radiolucent line at the last follow-up in 14.3% of the stemless TSA and 26.7% in the stemmed TSAs, although the difference did not reach statistical significance.^16^ Romeo et al^19^ did not observe radiolucent lines on follow-up imaging at any time point; however, they noted that this may be due to the relatively short follow-up period in comparison with studies that described this in stemless designs.^16,35^ Mariotti et al^17^ also reported no radiographic signs of loosening in the course of follow-up. By contrast, Wiater et al^18^ observed humeral radiolucency of >2 mm in two shoulders in the stemmed cohort and none in the stemless cohort, but no signs of loosening in either cohort, and concluded that stemless TSA demonstrated noninferiority regarding radiographic outcomes.

Previous studies have shown concern for radiolucent lines in some stemmed designs, although this was not shown to account for cortical bone fixation,^14,36^ and the actual incidence of adverse bone reaction was shown to be 2% at the 9-year follow-up using a specific stemless implant (Eclipse).^14^ This was also seen with stemmed (but not stemless) components in one of the included studies in the present analysis.^18^ A recent study by Pinto et al^15^ compared stemmed and stemless radiographic results in 79 patients and compared humeral head height, humeral head centering, humeral head medial offset, humeral head diameter, humeral neck angle, and lateral humeral offset. Their results yielded no difference in humeral head height, medial offset, lateral offset, head centering, and diameter.^15^ They did find that stemless implants showed improved restoration of native humeral neck angle at 0° for stemless compared with −3° for stemmed arthroplasties.^15^ A study by Razmjou et al^37^ examined stemmed and stemless designs for radiolucent lines and subsidence. Regarding subsidence, there was no statistically difference between components, but in one group of stemmed prostheses (Neer II, Smith & Nephew), 71% of the glenoids had radiolucent lines compared with 8% in the stemless (Total Evolutive Shoulder System, Biomet France SARL) design.^37^ Humeral radiolucent lines were more common with both stemmed implants (18% with Neer II, 8% with Bigliani-Flatow [Zimmer Biomet]) but with none of the Total Evolutive Shoulder System implants. An earlier study analyzed radiolucent lines and radiographic signs of humeral head subluxation and found no statistically significant difference between components, nor any clinical differences between patients with and without radiolucent lines or subluxation of their components.^38^ Although we did not quantitatively assess the radiographic results, the literature does not support a difference between stemmed and stemless humeral components at short-term follow-up, though future studies are warranted to confirm these results, establish their durability with longer follow-up, and determine the clinical relevance of such findings.

### Limitations

Although there were strengths to this study (inclusion of only the highest quality evidence of level I and II prospective randomized trials, consistent indication [PGHOA] for all patients in all included studies, strong statistical models, quantitative evaluation of multiple outcome indicators, and comparison of discrete adverse events), it was not without limitations. First, the results of any meta-analysis are limited by the availability of source studies in the literature, and at the time of writing, only four suitable studies could be identified for inclusion. Relatedly, the conclusions drawn from the synthesis of the available evidence are limited to the time frame of collection by the included studies. The four studies in the present analysis reported results for 2 years of follow-up; therefore, we cannot provide any additional conclusions about the durability of these results or about any differences that may arise at longer follow-up. Surgeons in the included studies were not blinded to the type of implant. This limitation, though often unavoidable in orthopaedic surgical trials, is reflected by the high risk of bias for the “blinding of participants and personnel” domain. Finally, a meta-analysis can only be conducted when there are comparable outcomes for quantitative comparisons, and we were therefore unable to analyze passive ROM instruments other than CS or additional specific radiographic outcomes.

## Conclusion

TSA with stemless versus stemmed humeral components was not associated with notable differences in functional and clinical outcomes. No difference was observed between stemmed and stemless designs in postoperative forward flexion, abduction, or external rotation. Similarly, there was no difference in change in forward flexion or external rotation. A markedly greater improvement in abduction was observed with stemmed TSA, likely due to the lower preoperative motion in the stemmed cohort in one of the studies. No differences were observed between stemmed and stemless designs in the rate of periprosthetic fracture or risk of revision.
